# The Role of FDG-PET in the Diagnosis and Monitoring of Large-Vessel Vasculitis

**DOI:** 10.1007/s11926-025-01204-w

**Published:** 2025-11-21

**Authors:** Alessandro Tomelleri, Corrado Campochiaro, Peter C. Grayson, Kaitlin A. Quinn

**Affiliations:** 1 Unit of Immunology, Rheumatology, Allergy and Rare Diseases, IRCCS San Raffaele Scientific Institute, Milan, Italy; 2 National Institute of Arthritis and Musculoskeletal and Skin Diseases, National Institutes of Health, 10 Center Drive, Building 10, 13C101, Bethesda, MD 20892 USA

**Keywords:** Giant cell arteritis, Takayasu’s arteritis, Large-vessel vasculitis, FDG-PET, Vascular imaging

## Abstract

**Purpose of this Review:**

Giant cell arteritis (GCA) and Takayasu’s arteritis (TAK) are the two main forms of large-vessel vasculitis (LVV), defined by inflammation of the aorta and its primary branches. Use of vascular imaging, including FDG-PET, has been increasingly incorporated into the assessment of patients with LVV. FDG-PET detects metabolic activity in the walls of the large arteries as a surrogate for vascular inflammation. In this article we review the use of FDG-PET to diagnose and monitor disease activity in different forms of LVV.

**Recent Findings:**

Use of FDG-PET to diagnose GCA by assessing vascular FDG uptake in the aorta and branch arteries is well-established. More recently, newer generation PET/CT scanners have also been used to assess metabolic activity in the cranial arteries, including the temporal arteries. In TAK, non-invasive angiography is used to assess for luminal damage at diagnosis, while FDG-PET can provide complementary information about whether active vascular inflammation is present. Recent studies have focused on the use of FDG-PET to monitor disease activity in LVV and the prognostic value of FDG-PET scans.

**Summary:**

Use FDG-PET in LVV remains an area of active ongoing research. While use at time of diagnosis in LVV has become well established, more studies are needed to evaluate the prognostic value of FDG-PET when monitoring disease activity in patients with LVV. Additional future directions for use of FDG-PET in LVV include employment of novel radiotracers, use of newer generation PET scanners, and incorporation into clinical trials to assess treatment response at the vascular level.

## Introduction

Large-vessel vasculitis (LVV) is defined by inflammation in the aorta and its primary branches, with giant cell arteritis (GCA) and Takayasu’s arteritis (TAK) being the two main forms of LVV [1]. Diagnosing LVV and assessing disease activity can be challenging, and use of non-invasive vascular imaging, including FDG-PET, is increasingly being used to aid in the assessment of patients with LVV (Table 1). Temporal artery biopsy has been the gold standard for the diagnosis of GCA, but is invasive, has a wide range of reported sensitivity from 39%−77%, and does not assess for large vessel involvement [2, 3]. In TAK, where histology is rarely available except in cases of surgical intervention, evaluation of these patients is dependent on vascular imaging.

**Table 1:** Use of FDG-PET to diagnose and monitor disease activity among different forms of large-vessel vasculitis in clinical practice

	GCA	TAK	Aortitis
Clinical symptoms	Typically associated with active disease	Can be difficult to differentiate damage vs active disease	May be asymptomatic
Time of Diagnosis	Useful as a diagnostic tool	Can be useful to assess vascular inflammation, in combination with non-invasive angiography to assess luminal damage	Can be helpful to assess vascular inflammation
Disease Activity Monitoring	Not advisable due to persistent vascular FDG-PET activity often observed in clinical remission of unclear clinical significance	Can be helpful after treatment escalation, particularly if uncertain about clinical response	Can be helpful to monitor vascular inflammation (especially if no corresponding vascular symptoms)
Prognostic value	Conflicting data about association between persistent PET activity and future relapse or angiographic progression	Limited value to predict relapse; FDG-PET activity may identify patients at risk for vascular damage	Not well assessed

*GCA* giant cell arteritis, *TAK* Takayasu’s arteritis

Positron emission tomography (PET) is a type of advanced molecular imaging. 18F-fluorodeoxyglucose (18F-FDG), a radiolabeled glucose analog, is the main radiotracer used in PET imaging in LVV [4]. Metabolically active cells utilize glucose more than other tissues, resulting in increased FDG uptake on FDG-PET. FDG-PET detects metabolic activity in the vessel wall, primarily reflecting glucose uptake by activated macrophages and other inflammatory cells infiltrating the arterial media and adventitia [4, 5].

This article aims to review the use of FDG-PET to diagnose and monitor disease activity in different forms of LVV.

## Image Acquisition Protocols

Guidelines for FDG-PET imaging protocols in LVV have been proposed [6, 7]. Patient preparation is important to reduce physiologic FDG uptake in normal tissues. Patients should consume a low carbohydrate diet for 24 h prior to FDG administration and fast for a minimum of 6 h to decrease physiologic uptake of glucose by the myocardium. Strenuous physical activities should be avoided for 24 h prior to the scan and patients should relax after FDG administration to minimize physiologic uptake in skeletal muscles. A blood glucose level below 7 mmol/L (126 mg/dL) is preferred, but < 10 mmol/L (180 mg/dL) is acceptable, as FDG uptake in tissue is reduced if serum glucose levels exceed this level. Weight based (e.g. 2–3 MBq/kg, 0.054–0.081 mCi/kg) or fixed-dose regimens (e.g. 10 mCi) have been used for FDG administration. Whole-body acquisition from the top of head down to lower extremities (at least mid-thigh, preferably below the knees) in the supine position with the arms next to the body is often recommended for patients with LVV, as patients with stenosing disease of the upper extremities or associated polymyalgia rheumatica (PMR) may be unable to hold their arms above the head for the duration of the scan [6, 7].

The interval between FDG administration and image acquisition should be at least 60 min, preferably 90–120 min, for adequate biodistribution of the radiotracer [6, 7]. Delayed imaging protocols lead to decreased blood pool activity and better visualization of the arterial wall and have been recommended in atherosclerosis [8]. One study comparing FDG-PET activity in patients with LVV at 60 min uptake time versus 120 min uptake time found that approximately 25% of patients had active vasculitis by FDG-PET only at the delayed uptake time of 120 min, and this was significantly associated with clinical disease activity [9]. Standardized uptake times are necessary to compare vascular uptake on FDG-PET in an individual patient over time.

FDG-PET images are frequently combined with low-dose computed tomography (CT) to allow for anatomical localization. In some cases, FDG-PET can be combined with contrast-enhanced CT for simultaneous acquisition of CT angiography (CTA) to assess for luminal damage (e.g. arterial stenosis or aneurysm) [6, 7]. FDG-PET can also be combined with magnetic resonance imaging (MRI) [10], although this is less frequently used due to lack of availability of scanners with PET/MRI capability. PET/MRI exposes patients to lower amounts of radiation, and may be a consideration, particularly for younger patients with TAK who require serial FDG-PET imaging.

## Image Interpretation in Large-Vessel Vasculitis

Interpretation of arterial FDG uptake in the context of suspected or confirmed vasculitis poses challenges. Interpretation of FDG-PET must be performed in a clinical context, with consideration for potential confounding factors that reduce scanner sensitivity such as concomitant use of glucocorticoids or other immunosuppressive therapies [11]. Differentiating arterial uptake secondary to atherosclerosis versus vasculitis can require clinical experience. FDG uptake that is substantially greater than background activity in the liver or blood pool, concentric uptake within the arterial wall, confluent uptake throughout arterial territories, or greater uptake in the arteries above versus below the diaphragm are all findings that are more suggestive of vasculitis than atherosclerosis [6].

Several metrics have been proposed to quantify arterial FDG uptake. Qualitative approaches involve visual inspection of arterial FDG uptake in specific arterial territories. Two composite scores have been proposed: the Total Vascular Score (TVS) [12] and the Positron Emission Tomography Vascular Activity Score (PETVAS) [13] (Table 2). TVS scores activity within individual arteries on a scale of 0-no uptake, 1-minimal uptake, 2-increased uptake, 3-marked uptake in the thoracic aorta, abdominal aorta, subclavian, axillary, carotid, iliac, and femoral arteries for a total summative score between 0–21. PETVAS uses a similar 0–3 grading system of arterial relative to liver uptake in 9 arterial territories (thoracic aorta, descending thoracic aorta, abdominal aorta, brachiocephalic, carotids, subclavians). The PETVAS territories were derived based on comparisons of arterial FDG uptake between patients with vasculitis versus atherosclerosis. PETVAS is easy to implement and has been used to demonstrate drug efficacy at the vascular level, most notably showing reduction in vascular inflammation in patients with GCA in response to treatment with tocilizumab [14].

**Table 2:** Scoring systems for vascular uptake on FDG-PET in large-vessel vasculitis

	Criteria	Advantages	Disadvantages
Subjective Assessment	Global impression by the reader as active or inactive vasculitis	Easy to implement, clinically actionable	Reliability among different raters may be poor in borderline cases
PET Vascular Activity Score (PETVAS)^13^	Uses a 0–3 grading system of arterial relative to liver uptake in 9 arterial territories (thoracic aorta, descending thoracic aorta, abdominal aorta, brachiocephalic, carotids, subclavians) for a total summary score of 0–27, with higher scores indicating greater activity	Easy to implement; territories derived based on comparisons of patients with atherosclerosis vs vasculitis; visual scoring system to assess for longitudinal change	Ceiling effect of scoring system (i.e. maximum score of 27); global summary score does not capture severe focal vasculitis well
Total Vascular Score (TVS)^12^	Scores activity within individual arteries on a scale of 0-no uptake, 1-minimal uptake, 2-increased uptake, 3-marked uptake in the thoracic aorta, abdominal aorta, subclavian, axillary, carotid, iliac, and femoral arteries for a total summary score of 0–21, with higher scores indicating greater activity	Easy to implement; visual scoring system to assess for longitudinal change	Ceiling effect of scoring system; global summary score does not capture severe focal vasculitis well
Standardized uptake values (SUV)	Semiquantitative metric that uses image contouring to determine SUVs in specific arterial regions. Mean or maximum SUV values can be reported in arterial territories	Offers greater precision than visual scores	Time-intensive to implement; confounding factors may affect FDG uptake in arterial tissues (e.g. age, body-mass index)^16^
Target to Background Ratios (TBRs)	Arterial SUV values normalized to SUV values in background tissue (e.g. liver, blood pool)	Offers greater precision; minimizes effect of factors that confound SUV measurements	Still susceptible to confounding factors that affect FDG distribution in both artery and background tissue (e.g. renal clearance)^16^
Vasculitis Activity Using MR and PET (VAMP) Score^10^	Combines FDG-PET uptake (expressed as the mean target-to-background SUV ratio across 12 arterial territories) with the presence of mural edema on T2-weighted MRI	Incorporates multimodal imaging	Specific to PET/MRI studies only; time intensive as uses SUVs and TBRs

Semiquantitative metrics have also been studied [15] (Table 2). These approaches rely upon image contouring to determine standardized uptake values (SUVs) in specific arterial regions. Mean or maximum SUV values can be reported in arterial territories. Arterial SUVs can be normalized to background tissue (e.g. liver, blood pool) SUV values and reported as Target to Background Ratios (TBRs). These approaches may offer greater precision when compared to composite scores like PETVAS but are more time-intensive to implement. Specific confounding factors may affect FDG uptake in arterial or background tissues and complicate interpretation of these values [16].

Several additional methods of PET assessment are noteworthy (Table 2). The Vasculitis Activity using MR and PET (VAMP) score is specific to PET/MRI studies and capitalizes on the use of multimodal imaging to assess LVV [10]. The VAMP score incorporates arterial FDG uptake as measured by semiquantitative approaches with visual inspection of abnormalities by vascular MR into a composite measure. Total lesion glycolysis (TLG) is a parameter that combines the volume and metabolic activity of FDG uptake at a tissue site. TLG has been proposed in LVV as a more comprehensive alternative to single-pixel measurements like maximum SUV value [17]. Artificial intelligence and radiomics will likely be increasingly adopted to optimize, automatic, and advance image interpretation in vascular PET [18].

## Role of Positron Emission Tomography in Giant Cell Arteritis

### Diagnosis

**Fig. 1:**
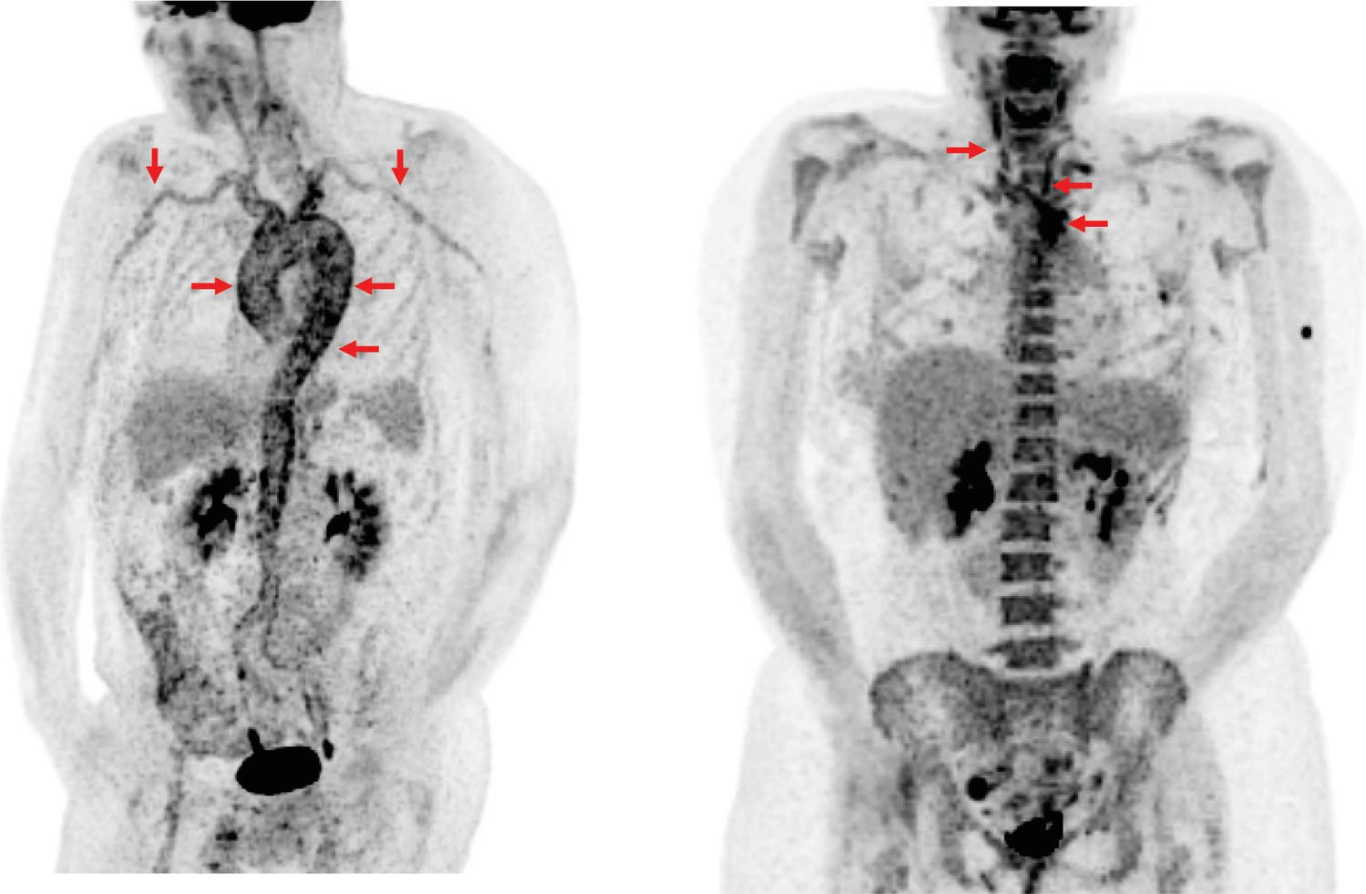
Representative FDG-PET scan images from a patient with giant cell arteritis (GCA) and a Patient with Takayasu’s Arteritis (TAK). A representative FDG-PET scan from a patient with newly diagnosed GCA is depicted (left image). The patient has severe vascular FDG uptake (radiotracer in black) diffusely throughout the aorta and bilateral subclavian/axillary arteries (red arrows). This pattern of diffuse vascular FDG uptake throughout the aorta is characteristic for GCA. A representative FDG-PET scan from a patient with newly diagnosed TAK is also shown (right image). The patient has severe vascular FDG uptake (black) in the aortic arch and bilateral carotid arteries. This focal vascular uptake throughout specific arterial territories is characteristic for TAK. *Images courtesy of the NIAMS vasculitis translational research program*

Use of FDG-PET to diagnose GCA by assessing vascular FDG uptake in the aorta and branch arteries is well-established **(**Fig. 1), (Table 1). In a meta-analysis from 4 pooled studies on the value of FDG-PET for diagnosis of GCA, FDG-PET demonstrated a high sensitivity (76%) and specificity (95%) to diagnose GCA, using clinical diagnosis as the reference standard [19–23]. Additionally, FDG-PET may be helpful to diagnose mimics of GCA in patients with atypical symptoms and one study identified clinically relevant incidental findings in 20% patients [23].

Previously, FDG-PET for the assessment of cranial arteries (e.g. temporal arteries, maxillary arteries) was not recommended due to limitations in the degree of resolution and proximity of cranial vessels to the severe physiologic FDG uptake of the brain [24, 25]. However, more recently, newer generation time-of-flight PET/CT scanners and new reconstruction methods have been used to assess metabolic activity in the cranial arteries [23, 26, 27]. Studies assessing the diagnostic performance of cranial PET/CT to assess uptake in temporal, occipital, maxillary, and vertebral arteries have shown sensitivities between 64% - 92% and specificity between 75% - 100%, depending on method of assessment (visual versus semiquantitative metrics) and reference standard (e.g. clinical diagnosis or temporal artery biopsy) [23, 26, 27].

Several challenges exist when using FDG-PET to assess for GCA. Active GCA must be differentiated from atherosclerosis, which can also cause arterial FDG uptake. However, FDG uptake in atherosclerosis often has a lower intensity, a “patchy” pattern (compared to a smooth linear pattern involving the aorta and its major branches in GCA), and frequently involves the iliofemoral arteries (compared to involvement of the aortic arch branch vessels in GCA) [6, 13]. Glucocorticoid use at the time of FDG-PET scan is an important consideration as it may confound assessment of vascular inflammation. One study randomized patients with GCA and active vasculitis by FDG-PET to have a repeat FDG-PET scan at day 3 or day 10 post initiation of prednisone. On day 3, all PET scans remained active, but by day 10 this number decreased to 36% active [11]. When possible, it is therefore recommended to obtain FDG-PET images within 72 h of initiating glucocorticoids [7]. However, other studies have shown a more limited effect of glucocorticoid use, with persistent FDG-PET activity observed despite longer duration of glucocorticoids [10, 28–30]. Importantly, initiation of glucocorticoids should not be delayed to obtain FDG-PET imaging, particularly in cases where there is strong clinical suspicion for GCA and concern for ischemia (e.g. vision loss).

Current rheumatology society guidelines support the use of FDG-PET to diagnose GCA. The American College of rheumatology recommends temporal artery biopsy first line to establish a diagnosis of GCA but recommends noninvasive vascular imaging for patients with suspected GCA and a negative temporal artery biopsy result. Potential diagnostic imaging modalities include use of FDG-PET [31]. EULAR also supports the use of FDG-PET for diagnosis in patients with suspected GCA [7]. Additionally, FDG-PET is incorporated into the most recent ACR/EULAR classification criteria for GCA [32]. Diffuse FDG uptake throughout the thoracic aorta without associated damage is an imaging pattern that can differentiate GCA from TAK [33].

### Monitoring Disease Activity

While FDG-PET is useful to establish a diagnosis of GCA, the utility of FDG-PET to monitor patients over time remains uncertain. In studies of patients with GCA who underwent repeat FDG-PET scans, the amount of vascular uptake on FDG-PET significantly decreased over time in GCA [34, 35]. However, often patients with GCA continue to have some level of ongoing vascular inflammation by FDG-PET later in the disease course, frequently during periods of clinical remission [13, 36–38]. Whether ongoing FDG-PET activity represents subclinical vascular inflammation (versus vascular remodeling or atherosclerosis) remains uncertain, but histologic studies conducted in later-stage GCA have shown that subclinical vascular inflammation during clinical remission often occurs [39–42].

The association of persistent FDG-PET activity with longitudinal outcomes in GCA is not well established. Patients with GCA often develop disease relapses. An association between FDG-PET activity and future relapse risk has been reported in some studies [13, 43], while several other studies have not demonstrated a clear association [35, 38, 44–46]. In particular, whether obtaining a repeat FDG-PET scan after treatment discontinuation is helpful to predict future relapse remains unknown, but preliminary data from some studies suggest this may be an important area of future investigation [38, 47].

Patients with GCA can also develop vascular damage, including aneurysm or arterial stenoses. Studies have reported an association of baseline vascular FDG uptake and subsequent arterial damage in GCA [48, 49], with one prospective cohort study of 106 patients demonstrating those with an active FDG-PET scan had a higher risk for development of thoracic aortic aneurysms [49]. In another prospective, longitudinal study in GCA, no arterial territories with baseline FDG-PET activity developed new or worsened damage by non-invasive angiography over a median 1.6-year follow-up period [50]. In studies where patients have undergone serial FDG-PET scans, an association with persistent FDG-PET activity at time of clinical relapse and the development of thoracic aortic aneurysms was reported in one cohort [51], while another cohort did not show any association with FDG-PET activity during clinical remission and subsequent vascular damage [38].

Overall, monitoring of patients by serial FDG-PET scans is not routinely recommended in GCA, due to uncertainty about the clinical significance and prognostic value of persistent FDG-PET activity, and concerns this may in turn lead to unnecessary radiation, additional cost, and prolonged immunosuppression [7, 31] (Table 1). However, repeat FDG-PET scans can be considered at time of clinical relapse, particularly in cases where there is uncertainty about disease activity (i.e. non-specific symptoms with increased acute phase reactants) [38].

Response to specific treatments can also be assessed by FDG-PET. A reduction in vascular FDG uptake has been observed with tocilizumab treatment [14, 29, 37, 47]. A recent study reported a greater reduction in FDG-PET activity in patients with GCA treated with tocilizumab compared to glucocorticoid monotherapy [38]. Incorporation of FDG-PET in future clinical trials in GCA may be useful to help quantify response to a specific treatment at the vascular level.

## Role of Positron Emission Tomography in Takayasu’s Arteritis

While the classification criteria for TAK have been recently updated [52], establishing a diagnosis in clinical practice remains particularly challenging, especially in the early phases when systemic inflammatory symptoms may precede overt vascular involvement [53]. Over the past two decades, FDG-PET has emerged as a valuable tool in the diagnostic workup of TAK, adding a functional dimension to anatomical modalities such as magnetic resonance angiography (MRA), computed tomography angiography (CTA), and catheter-based angiography (Fig. 1). Although not formally incorporated into classification criteria for TAK, FDG-PET is acknowledged as one of the acceptable imaging modalities to fulfill the requirement of demonstrating LVV [52]. Its ability to reveal inflammation prior to structural changes provides a key advantage over conventional imaging, especially in capturing the so-called “pre-stenotic phase” of TAK, which may be missed by MRA or CTA. Although comparative studies specifically designed for diagnostic purposes are lacking, FDG-PET has demonstrated utility in identifying early disease and subclinical vascular involvement [13, 54] (Table 1).

The whole-body field-of-view of FDG-PET allows for assessment of multiple vascular territories, frequently revealing unexpected sites of involvement. Additionally, FDG-PET can help exclude alternative diagnoses such as infection, lymphoma, or paraneoplastic syndromes that may clinically mimic TAK but display distinct metabolic patterns [55]. Two large meta-analyses have evaluated the diagnostic performance of FDG-PET in TAK, reporting pooled sensitivities of 84% and 76% and specificities of 98% and 93%, respectively [56, 57]. However, these data were primarily derived from broader LVV cohorts, limiting their direct applicability to TAK. In particular, comparisons with GCA may be misleading and underscore the need for TAK-specific diagnostic evaluations.

The modest diagnostic accuracy of FDG-PET in TAK may reflect the limitations of reference standards rather than the imaging modality itself. In early TAK, FDG uptake may reveal active inflammation before structural abnormalities develop, while in chronic stages, vascular damage may persist in the absence of inflammation, resulting in apparent false negatives. Conversely, increased FDG uptake in clinically inactive patients may indicate subclinical or evolving disease. These discrepancies highlight the capacity of FDG-PET/CT to detect inflammation beyond what is captured by traditional diagnostic frameworks [50].

Although FDG-PET may not outperform other modalities in inactive or fibrotic TAK, it provides a key advantage in detecting early disease, particularly in young patients with nonspecific symptoms in whom conventional imaging may be unrevealing. Importantly, FDG-PET interpretation must always be contextualized within the broader clinical picture, as other vasculitides (e.g., Behçet’s disease, relapsing polychondritis, IgG4-related disease) can present with overlapping features [58]. Glucocorticoid therapy substantially impacts FDG-PET sensitivity; although most evidence stems from GCA, reduced FDG uptake with increasing steroid exposure likely applies to TAK [11, 28]. Diagnostic performance is highest when FDG-PET is performed before or within a few days of treatment initiation.

In addition to its clinical utility, FDG-PET has been proposed as a tool for standardizing patient selection in clinical trials [59]. A recent international survey among physicians experienced in TAK management showed that 84% routinely use FDG-PET to support the diagnosis, reflecting its established role in clinical practice. More recently, FDG-PET combined with MRI/MRA has gained attention as a radiation-sparing alternative to CT in LVV, including TAK. Although most current evidence relates to disease monitoring, its role in diagnosis is likely to expand, particularly given its capacity to integrate metabolic and structural assessment in a single session [60, 61].

Beyond diagnosis, FDG-PET plays an important role in the longitudinal monitoring of TAK, particularly given the known limitations of conventional tools. Indeed, clinical assessment, acute-phase reactants, and traditional indices such as ITAS2010 often fail to accurately reflect vascular inflammation [62, 63]. In this context, FDG-PET has gained attention as a modality capable of directly visualizing arterial wall inflammation, providing a functional complement to anatomical imaging and clinical evaluation (Table 1).

In the original PETVAS study, which included 26 patients with TAK, PETVAS scores were significantly higher in those with clinically active disease than in those in remission [13]. However, concordance with clinical assessment was suboptimal: 48% of patients with TAK in clinical remission had FDG-PET scans interpreted as active, and one patient had a PETVAS ≥ 20 (the threshold that best distinguished clinically active LVV). Moreover, while PETVAS correlated moderately with ESR, CRP, and fibrinogen during active disease, these associations disappeared in remission.

Subsequent studies provided further insights into the performance of PETVAS in TAK. In a large single-center cohort, PETVAS was significantly associated with clinical disease activity, but its overall accuracy in distinguishing active from inactive disease remained moderate [45]. FDG uptake also persisted in a substantial proportion of scans performed during clinical remission, even under treatment. A longitudinal FDG-PET/MRI study confirmed that although PETVAS declined with therapy, it did not significantly differentiate between active and inactive TAK [10].

To address these limitations and reduce the risk of overinterpreting isolated findings, the Takayasu Arteritis Integrated Disease Activity Index (TAIDAI) was developed. This composite score integrates clinical symptoms, FDG-PET findings, and acute-phase reactants in a hierarchical structure that prioritizes clinical judgment and validates imaging or laboratory abnormalities only when they support a clinical suspicion of activity. In a prospective multicenter cohort of 96 patients with TAK, TAIDAI showed strong correlation with PETVAS and physician global assessment and was responsive to treatment escalation. Notably, a TAIDAI score of 0 was observed in over 90% of patients in clinical remission, highlighting its potential utility in clinical trials and as a treat-to-target endpoint in practice [64].

Beyond composite indices, a hybrid imaging approach has also been explored. In a prospective FDG-PET/MRI study, the VAMP score outperformed PETVAS in identifying active disease and showed strong correlations with CRP and global disease activity [10]. However, in TAK specifically, neither PETVAS nor VAMP reliably distinguished active from inactive disease. This may reflect the focal nature of inflammation in TAK and the confounding impact of chronic vascular changes, which can blur the distinction between activity and damage on imaging.

The discrepancy between metabolic and structural imaging in TAK is underscored by a recent pediatric FDG-PET/MRI study [65]. Among 17 patients, most in clinical remission, FDG-PET and MRI findings were often discordant: only 13% of abnormal segments showed concordant changes, while the majority (59.6%) were PET-positive and MRI-negative. A smaller subset showed MRI abnormalities without FDG-PET uptake. FDG uptake and metabolic volume were significantly higher in concordant lesions, especially in the ascending aorta. These results suggest that FDG-PET and MRI capture distinct aspects of vascular pathology and support their combined use in disease monitoring.

The prognostic value of FDG-PET for predicting clinical relapses in TAK remains limited. In the original PETVAS study, a PETVAS ≥ 20 during clinical remission was associated with an increased risk of subsequent relapse in the overall LVV cohort, though no TAK-specific analysis was reported [13]. A larger single-center study explored this question more directly: in patients with TAK undergoing FDG-PET during clinical remission, PETVAS was not significantly associated with subsequent relapses [45]. The AUC for relapse prediction in TAK was 0.59, and a PETVAS ≥ 9 yielded only 52.6% sensitivity and 65.8% specificity. These results suggest that although FDG-PET may detect persistent vascular inflammation, its standalone performance for predicting flares remains suboptimal.

Beyond relapse prediction, FDG-PET has also been investigated as a potential tool for identifying patients at risk of developing or worsening vascular damage. In a large prospective LVV cohort including individuals with TAK, baseline FDG uptake was frequently observed, yet new or progressive angiographic damage over a median follow-up of 1.6 years was uncommon [50]. Most FDG-PET-active segments remained stable, and FDG-PET activity showed low positive predictive value overall. However, in TAK patients, arterial segments that developed damage were significantly more likely to have shown baseline FDG uptake, with an increased risk when uptake coexisted with mural thickening or edema on anatomical imaging.

Two additional observational studies focusing exclusively on TAK further explored the predictive value of FDG-PET for vascular damage [66, 67]. In both cohorts, higher baseline PETVAS scores were observed in patients who later developed angiographic progression. PETVAS thresholds associated with damage evolution were identified (≥ 15 in one study and ≥ 10 in the other) with moderate sensitivity and high specificity. While these findings suggest that increased FDG-PET activity may help identify patients at risk of damage, segment-level analysis was not formally reported, and FDG uptake alone lacked sufficient specificity to reliably predict progression.

Taken together, these data suggest that while baseline FDG-PET activity may help identify patients at increased risk of vascular damage, its predictive accuracy remains modest. PET-derived metrics such as PETVAS or regional SUV ratios should be interpreted in conjunction with anatomical imaging and clinical context. Multimodal imaging strategies are likely to be most informative for risk stratification and long-term monitoring in TAK.

Despite several advantages, FDG-PET is not without limitations. Physiological FDG uptake in large arteries, particularly in older individuals or those with metabolic syndrome, may lead to false positives, while diffuse low-grade uptake can be difficult to interpret in the absence of overt clinical features [6]. The lack of universally accepted scoring thresholds and the subjectivity inherent in visual assessment contribute to interobserver variability. Standardization of acquisition protocols, reader training, and availability of validated reference atlases are essential to improve reliability and reproducibility. Accessibility and cost also remain important barriers and the use of FDG-PET may be limited by reimbursement policies and infrastructure constraints. Although radiation exposure is modest, it must be considered in younger patients and in situations where repeated imaging is anticipated. In childhood-onset TAK, MRA remains the preferred modality due to its safety profile and widespread availability [68].

## Role of Positron Emission Tomography in Other Forms of Large-Vessel Vasculitis

Sir William Osler reportedly once said, “there is no disease more conducive to clinical humility than aneurysm of the aorta.” One hundred years later, the sentiment still holds true. The challenge is that inflammation and structural disease limited to the aorta is often asymptomatic but can be catastrophic at presentation. Because of the sensitivity of FDG-PET to detect pathology prior to structural damage, it is increasingly used for inflammatory or infectious indications and may have a role in cases where there is fever or inflammation of unknown origin [69]. Additionally, FDG-PET can be used to categorize disease extent during the initial evaluation of complex systemic inflammatory diseases. In these settings, severe FDG uptake in the aorta can often be discovered incidentally. Patterns of organ system involvement by FDG-PET provide important diagnostic clues. In sarcoidosis, FDG uptake can define extra thoracic involvement of disease and reveal associated aortitis, the extent of lymphadenopathy, cardiac and neurologic involvement [70]. In Erdheim Chester Disease, hypermetabolic lesions in the skeleton, retroperitoneal space, around the kidneys, and diffusely through the descending aorta are characteristic [71]. In IgG4-related disease, PET abnormalities involving the salivary and lacrimal glands, sinonasal cavities, lung parenchyma, pancreas, retroperitoneum, and lymph nodes can accompany vascular FDG uptake which often involves the abdominal aorta extending into the iliofemoral arteries [72]. Use of FDG-PET to diagnose and to monitor treatment response is often considered, especially in cases where aortitis is incidentally discovered in an asymptomatic patient (Table 1).

## Novel Radiotracers and New Technology

While FDG is a valuable tool to assess inflammatory diseases, FDG is fundamentally a maker of increased glycolysis rather than a direct measurement of vascular inflammation. Differentiating active inflammation from smooth muscle remodeling within a previously inflamed artery remains a tremendous unmet need. Ongoing efforts to develop novel radiotracers that target specific cell populations have led to approved indications for a number of novel radiotracers [73]. Radiotracers that bind to IL-2 receptors, CD4, or CD8 molecular can be markers for T cell populations. Macrophages can be visualized by tracers that target folate receptors or somatostatin. Fibroblast activation protein (FAP) may be a marker for activated fibroblasts, although hematopoietic cells also express FAP [74, 75]. Unlike their use to visualize solid tumors, differentiating uptake of novel radiotracers that target specific hematopoietic cells within the vascular wall versus the adjacent blood pool is a complicating factor in vascular PET. For now, these agents remain investigational in LVV, and 18FDG continues to be the standard tracer in both clinical care and research.

In addition to novel radiotracers, technical advancements in PET imaging continue to improve the sensitivity of imaging. Integration of PET with MRI improves accuracy of localizing PET abnormalities within the vasculature and minimizes radiation exposure. Long-axial-field-of-view (LAFOV) PET systems improve spatial resolution and enable the detection of metabolic abnormalities in smaller arteries, including branches of the external carotid artery that are commonly affected in GCA [76]. These technical improvements reduce scan time, radiotracer dose, and radiation exposure, making it easier to incorporate PET imaging into a clinical workflow.

## Conclusion

The past decade has seen major advancement in clinical care and research for patients with LVV. Several randomized controlled trials have demonstrated efficacy of various medications in LVV [77–80]. Use of PET in the coming years will be important to understand the nuances of effective therapy. It is anticipated that characterization of disease activity at the clinical, laboratory, and imaging level will continue to refine definitions of disease states and inform the scope of treatment efficacy.

## Key References

- Pugh, D, et al*.* (18)F-FDG-PET/MR imaging to monitor disease activity in large vessel vasculitis. Nat Commun. 2024.
- Sammel AM, et al. Repeated Cranial and Large-Vessel Positron Emission Tomography/Computed Tomography Scans and the Association With Structural Aortic Disease in Giant Cell Arteritis: A Five-Year Observational Study. ACR Open Rheumatol. 2025.
- Muratore F, et al. Treatment of giant cell arteritis with ultra-short glucocorticoids and tocilizumab: results from the extension of the TOPAZIO study. Rheumatology (Oxford). 2025.
- Moreel L, et al. Association Between Vascular 18(F)-Fluorodeoxyglucose Uptake at Diagnosis and Change in Aortic Dimensions in Giant Cell arteritis. 2023.
- Quinn KA, et al. Association of 18F-Fluorodeoxyglucose-Positron Emission Tomography Activity with Angiographic Progression of Disease in Large Vessel Vasculitis. Arthritis Rheumatol. 2023

## Data Availability

No datasets were generated or analysed during the current study.
